# Evidences for Cooperative Resonance-Assisted Hydrogen Bonds in Protein Secondary Structure Analogs

**DOI:** 10.1038/srep36932

**Published:** 2016-11-16

**Authors:** Yu Zhou, Geng Deng, Yan-Zhen Zheng, Jing Xu, Hamad Ashraf, Zhi-Wu Yu

**Affiliations:** 1 Key Laboratory of Bioorganic Phosphorous Chemistry and Chemical Biology (Ministry of Education), Department of Chemistry, Tsinghua University, Beijing 100084, P. R. China; 2 School of Chemistry and Chemical Engineering, Qingdao University, Qingdao 266071, P. R. China

## Abstract

Cooperative behaviors of the hydrogen bonding networks in proteins have been discovered for a long time. The structural origin of this cooperativity, however, is still under debate. Here we report a new investigation combining excess infrared spectroscopy and density functional theory calculation on peptide analogs, represented by *N*-methylformamide (NMF) and *N*-methylacetamide (NMA). Interestingly, addition of the strong hydrogen bond acceptor, dimethyl sulfoxide, to the pure analogs caused opposite effects, namely red- and blue-shift of the N−H stretching infrared absorption in NMF and NMA, respectively. The contradiction can be reconciled by the marked lowering of the energy levels of the self-associates between NMA molecules due to a cooperative effect of the hydrogen bonds. On the contrary, NMF molecules cannot form long-chain cooperative hydrogen bonds because they tend to form dimers. Even more interestingly, we found excellent linear relationships between changes on bond orders of N−H/N−C/C = O and the hydrogen bond energy gains upon the formation of hydrogen bonding multimers in NMA, suggesting strongly that the cooperativity originates from resonance-assisted hydrogen bonds. Our findings provide insights on the structures of proteins and may also shed lights on the rational design of novel molecular recognition systems.

Hydrogen bonds play vital roles in the formation of secondary structures of proteins, such as *α*-helix and *β*-sheet^1^. A special property of these hydrogen bonds is their cooperativity, typified by the extra energy gain upon extension of the hydrogen bond units, (N−H···O = C)_*n*_. It was first reported by Salemme in 1982, and since then has been studied by many laboratories^2^,^3^,^4^,^5^,^6^,^7^,^8^,^9^. Several important protein behaviors, including the aggregation of *β*-amyloid peptides that may lead to Alzheimer’s disease or mad-cow disease, are attributed to this cooperativity^10^. The construction of some functional materials also took advantage of this special effect of hydrogen bonds^11^.

The origin of the cooperativity has been investigated by calculation for years and different opinions were proposed^12^. A classical hypothesis is that resonance-assisted hydrogen bonds (RAHBs) participate in the formation of protein secondary structures^13^,^14^,^15^. In this hypothesis, the lone pair electrons on the nitrogen atom and the *π* bond of the carbonyl group in peptide bonds would resonate to an enol-like structure as shown in Fig. 1. Some biochemical processes are explained following this hypothesis. For example, the electron transfer chain between two heme rings in cytochrome *b*_561_ is clarified as serials of RAHBs^16^. However, some other works attribute the cooperativity to long-range electrostatic interactions^17^, van der Waals interaction^18^, *σ-*orbital interaction^19^, electronic *σ-*framework rearrangement^20^ or charge interaction^21^. Unfortunately, there has been no experimental evidences reported on RAHB in proteins and protein analogs, to the best of our knowledge.

We tried to address the issue by taking the two molecules shown in Fig. 1 (NMF and NMA) as protein secondary structure analogs^22^,^23^,^24^,^25^. Another molecule used in our study is dimethyl sulphoxide (DMSO), which is known to interact strongly with proteins/peptides^26^,^27^ and to be a good water-miscible solvent to improve the solubility of hydrophobic compounds in water^28^, so that is applied widely in biochemistry^29^,^30^. In addition, DMSO can also work as cryo-protectant^31^,^32^, cell fusogen^33^,^34^,^35^, cell differentiation inducer^36^,^37^, and membrane/skin permeation enhancer. To this end, the self-association structures of both NMF and NMA have common hydrogen bonds in the form of N−H···O = C, the same as in proteins^38^,^39^. Studies on the interaction between DMSO and amides indicated that this aprotic solvent can break the N−H···O = C hydrogen bond between amide molecules by forming stronger N−H···O = S hydrogen bond^40^,^41^,^42^. Thus, the infrared vibration spectroscopic properties of the two binary systems, NMF-DMSO and NMA-DMSO, are expected to be similar. But unexpectedly, we observed opposite results, which indicate different hydrogen bonding modes between NMF and NMA. The calculating results confirm this conclusion and suggest the origin of the cooperativity of hydrogen bonds is RAHB.

## Results

### IR spectra of N−H stretching modes

Attenuated total reflection Fourier transform infrared spectroscopic (ATR-FTIR) technique was employed to get original infrared spectra. As shown in Fig. 2A, red shift (19.1 cm^−1^) of the N−H stretching infrared absorption was recorded when introducing DMSO into NMF, while blue shift (5.9 cm^−1^) was observed in the case of NMA as in Fig. 2C. It should be noted that deuterated DMSO (DMSO-*d*_6_) was used in the measurements to avoid overlap of methyl C−H stretching vibration peaks between amide and DMSO.

The infrared spectral data have been analyzed by excess spectroscopy^43^,^44^,^45^,^46^ as shown in Fig. 2B,D. Clearly, we see opposite features of the excess spectra in the N−H stretching region. In NMF-DMSO-*d*_6_ system, for each concentration, the negative peak is on the high wavenumber side (around 3380 cm^−1^) and the positive peak is on the low wavenumber side (about 3260 cm^−1^). In NMA-DMSO-*d*_6_ system, the feature is just reversed: the negative (or positive) peak is on the low (or high) wavenumber side. These results imply the apparent different interaction modes of the two analog molecules in interacting with DMSO. Furthermore, both positive and negative peak positions in the excess spectra are relatively fixed, suggesting that the self-associating complex of NMF and NMA, as well as the newly formed complexes between NMF/NMA and DMSO, are stable^45^.

### IR spectra of C = O stretching modes

As for C = O bond, which is the main proton acceptor group in the two analogs, the ordinary and excess IR spectra are shown in Fig. 3. The peak positions of C = O stretching vibration mode in both NMF and NMA are blue-shifted with adding DMSO-*d*_6_. This is expected, because C = O groups only participate in N−H···O = C hydrogen bonds between the amide molecules, the blue-shifts indicate that C = O bonds are strengthened after dissociation. No opposite changes are seen as in the case of *ν*(N−H).

The excess IR spectra in the C = O stretching vibration region are shown in the lower panels in Fig. 3. As can be seen in the figure, the positions of both positive and negative peaks are fixed, in agreement with the results shown in Fig. 2. A feature worth of reminding is that multiple negative peaks are seen in Fig. 3D, which suggests that populations of different self-association structures of NMA would decrease with increasing concentration of DMSO-*d*_6_. On contrast, the excess peaks of NMF in Fig. 3B are similar to a simple two-state transformation situation^45^.

### Optimized amide complexes by quantum chemical calculations

The hydrogen bonds involving N−H are classified as red-shifted hydrogen bonds^47^,^48^, thus the red- and blue-shift of *ν*(N−H) in the two binary systems indicate the strengthening and weakening of the hydrogen bonds. To reveal the different relative energy relationships of self-associating amides and amide-DMSO complexes in the two systems, we turned to quantum chemical calculations. Selected average hydrogen bond energy *E* and configurations of representative complexes are shown in Fig. 4. For the self-association of NMA, due to the steric effect of the methyl groups, the associating complexes tend to choose linear configuration. Continuous increase in the absolute value of *E* from NMA dimer to hexamer implies the cooperativity of the related hydrogen bonds. In the case of NMF, two conformers, *cis*- and *trans*-NMF, exist in pure liquid. According to literature^49^ and our experiment (Figure S3), we consider only the self-associates of *trans*-NMF and *cis*-NMF-*trans*-NMF dimers.

As can be seen in Fig. 4, the energy level of NMF-DMSO complex is between the trimer and dimer of NMF. Because we observed red-shift of *ν*(N−H) upon addition of DMSO into NMF, the dominant species in the binary mixtures are either monomers or non-cooperative dimers. This is in agreement with the conclusions in literature^50^. In the case of NMA, the energy level of NMA-DMSO complex is also between the trimer and dimer of NMF. However, because blue-shift of *ν*(N−H) upon addition of DMSO into NMA was observed, we conclude that the dominant species in the binary mixtures are oligomers of NMA, larger than dimers. Very importantly, these oligomers are cooperative, namely there is an extra energy gain upon formation of longer hydrogen bonding chains. Further, the oligomers of NMA from hexamer (or even larger ones) to trimer would dissociate upon addition of DMSO to NMA, which could be the reason of multiple-negative-peak feature in the excess spectra of Fig. 3D. It is noteworthy that the presence of cooperativity in the hydrogen bond network of NMA makes it a better model to peptides/proteins than NMF.

Taking the picture of Fig. 4 in mind, we may predict that diluting NMF and NMA with an inert solvent, for example CCl_4_, will result in blue shift of *ν*(N−H), because the diluting processes in both cases would cause the weakening of the hydrogen bonding interaction among the amide molecules. Our ATR-FTIR experiments supported the prediction (Figure S4). Likewise, the parallel blue shifts of *ν*(C = O) shown in Fig. 3C can be explained easily, as the diluting processes of the two amides by DMSO only concern with the breaking of the carbonyl-involved hydrogen bonds. The hydrogen bonds between the carbonyl groups and the methyl groups of DMSO, if there are any, are very weak and can be ignored.

**Figure 1:**
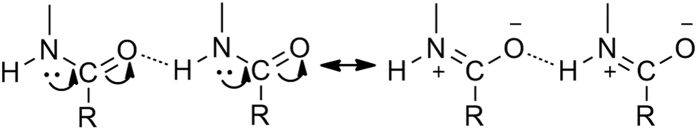
The RAHB between model molecules NMF (R = H) or NMA (R = CH_3_).

**Figure 2:**
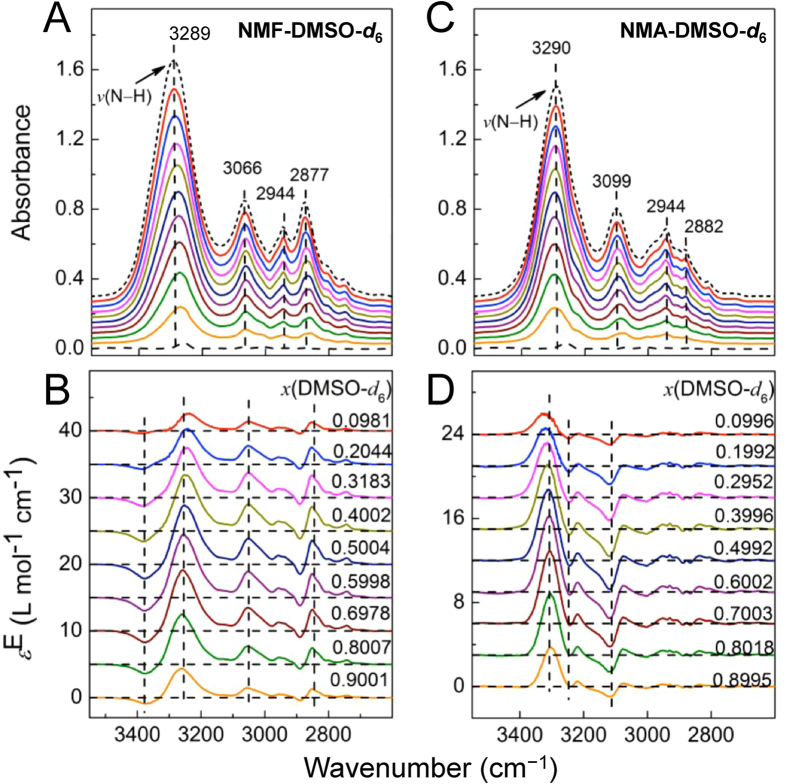
The IR (**A,C**) and excess IR (**B,D**) spectra of NMF-DMSO-*d*_6_ (**A,B**) and NMA-DMSO-*d*_6_ (**C,D**) systems in the range of N−H and C−H stretching vibration region. The short-dashed line and dashed line in (**A,C**) depict the spectra of pure NMF/NMA and DMSO-*d*_6_. The vertical dashed lines are used to guide eyes. The horizontal dashed lines in (**B,D**) are relative baselines for respective excess IR spectra. From top to bottom in (**A,C**) the mole fraction of DMSO-*d*_6_ increases from 0 to 1. The precise mole fractions are labeled in (**B,D**).

**Figure 3:**
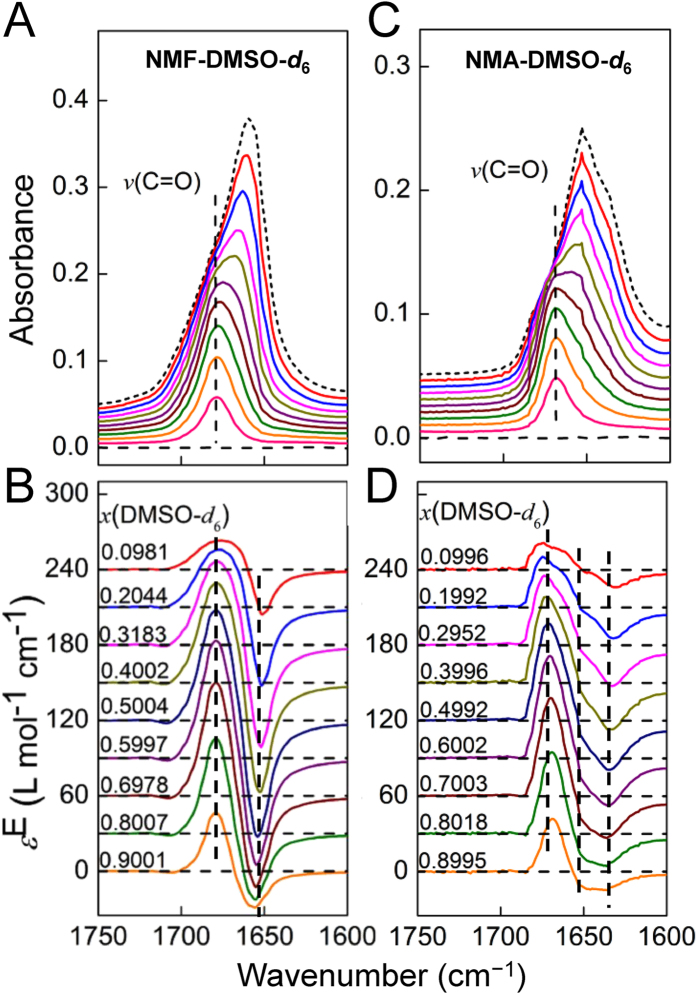
The IR and excess IR spectra of NMF/NMA-DMSO-*d*_6_ system in the C = O stretching vibration region. See the caption of Fig. 2 for other explanations.

Now we address the important issue, the origin of cooperativity of hydrogen bonds, by taking NMA as the model system. Following the discussion above, we learnt that the bond strength of N−H or C = O in the non-cooperative case (NMA-DMSO and NMA-CCl_4_ mixtures) is stronger than that in the cooperative hydrogen bonding associates (pure NMA). This means that the cooperative process weakens the strength of these bonds, which is in line with the resonance effect showing in Fig. 1. Inspired by this, we calculated the average bond orders (BOs) of C = O, N−H and C−N in different NMA self-associating complexes using the natural bond orbital (NBO) analysis method^51^. Not surprisingly, resonance between peptide bond structure and enol-like structure has already existed in NMA monomer, where the bond orders of C = O, N−H and C−N bonds are 1.658 (<2), 0.794 (<1), and 1.197 (>1), respectively. What surprises us is the perfect linear relationships between the average hydrogen bond energies and the changes on bond orders of the three covalent bonds forming the resonance structures, as shown in Fig. 5.

In Fig. 5, the C = O and N−H bond orders are positively related to the average hydrogen bond energy, while the C−N bond order is negatively related. This means that the gain in the hydrogen bond energy accompanies with the increase in the bond order of C−N and the decreases in the bond orders of C = O and N−H. The perfect linearity strongly suggests that the cooperativity of hydrogen bonds originates from the RAHBs. The charge-redistributed enol-like structure would strengthen the hydrogen bond between N−H and O = C.

## Discussion

By comparing the behavior of two similar molecules, *N*-methylformamide and *N*-methylacetamide, in forming hydrogen bonds with an aprotic molecule, dimethylsulphoxide, we have been fortunate to observe opposite shifting of the N−H stretching absorption bands. This allows us to state that *N*-methylacetamide is a better molecular model in studying peptide bonds, particularly when the issue of cooperative hydrogen bonds in proteins/peptides is under concern. Frequently, formamide and *N*-methylformamide are used as the model molecules. For example, Dannenberg^12^ and Wu’s groups^17^ once used formamide to study the cooperativity in amide hydrogen bonding chains. Although it is almost perfect to perform quantum chemical calculations using the small molecule, the two N−H bonds in the molecule provide too many possibilities in forming hydrogen bonds with another molecule. As a result, it is virtually impossible to expect the simple linear arrangement of the molecule in a real sample. This could be an important reason that no experimental work has been reported in testing these studies. The replacement of one N−H bond by N−CH_3_ reduces such possibilities. But the derived molecule, *N*-methylformamide, is still not perfect. On the one hand, the carbonyl groups in real peptides connect with alkyl groups, while in *N*-methylformamide it bonds with a hydrogen atom. This gives the C−N bond more freedom to rotate, causing *cis-* and *trans-*conformers. On the other hand, the *cis-*conformers would terminate the extension of hydrogen bond chains (see SI for details). Different from these two molecules, *N*-methylacetamide has been shown to be the best model molecules among these derivatives. This is because the repulsion between the two methyl groups allows only *trans-*conformers of this molecule and thus favors the extension of hydrogen bond chains.

Regarding the origin of the cooperativity of the hydrogen bonds in proteins/peptides, our work suggests that it is the resonance of the amide. The perfect linear relationships between the interaction energy and bond order changes of the three key covalent bonds, C = O, N−H and C−N, strongly support this conclusion. Zhao and Wu used quantum chemical calculations to study the hydrogen bond energies of formamide self-associations^17^. They found that introduction of a medium (methanol) to the hydrogen bonding network in gas phase markedly reduced the cooperative energy, and thus proposed that the main reason of cooperativity is electrostatic interaction. Qualitatively speaking, we think the resonance-origin and electrostatic interaction-origin is not contradictory. This is because that resonance of the amide structure will cause redistribution of charges in the concerned molecules and thus will exert an influence on the electrostatic interactions. Similarly, we can also use the resonance model to explain the idea of van der Waals interaction-origin proposed by Hua *et al*.^18^, because the charge redistribution from the enol-like resonance structures would put an effect on the dipole moments of the molecules and thus will affect the van der Waals interaction, as we just explained. Actually, the amide molecules discussed here are all neutral. Under this circumstance, diploe-dipole interaction and electrostatic interaction are the same in nature.

To sum up, we report the experimental evidences on the cooperativity of hydrogen bonds in NMA in this work. Compared with other amides, such as the popular formamide and *N*-methylformamide, only *N*-methylacetamide prefers to forming long chain structure, which makes it a better model molecule to peptides/proteins. We discovered perfect linear relationships between the average changes on the bond order of C = O, N−H, and C−N and the average hydrogen bond energies in various associates of NMA, which are consistent with the bond strength changes revealed by infrared experiments. We thus conclude that the origin of the cooperativity is the resonance of the peptide bonds in the hydrogen bonding network of proteins. The findings of this work may inspire the understanding of some biochemical processes and rational design of functional materials using cooperative hydrogen-bond strategies.

## Methods

### Chemicals

DMSO-*d*_6_ (99.8 atom% D) was purchased from Armar Chemicals. *N*-methylformamide (NMF), *N*-methylacetamide (NMA), and *N*,*N*-dimethylformamide (DMF) (99%) were from J&K Scientific. CCl_4_ (99.5%) was from Beijing Chemical Works. The samples were used without further purification. For the preparations of the binary mixtures, a series of NMF−DMSO-*d*_6_, NMA−DMSO-*d*_6_, and DMF−DMSO-*d*_6_ binary systems were prepared by weighing.

### FTIR Spectroscopy

ATR-FTIR spectra over the range from 4000 to 650 cm^−1^ were collected at room temperature using a Nicolet 5700 FTIR spectrometer, equipped with an MCT detector. Two ATR cells made of trapezoidal ZnSe/Ge crystals were used in the experiments. ZnSe crystal with an incident angle of 45° and 12 reflections was used to examine the stretching bands of N−H and C−H in amides and C−D in DMSO-*d*_6_. The Ge crystal with an angle of 60° and 7 reflections was used to examine the strong stretching absorption band of C = O. Spectra were recorded with a resolution of 2 cm^−1^, a zero filling factor of 2, and 32 parallel scans. The refractive indexes of solutions were measured with a refractometer at room temperature, around 26 °C. The formulas suggested by Hansen^52^ were used to do the ATR corrections.

### Excess Infrared Spectroscopy

The theory of excess infrared spectroscopy has been described in detail elsewhere^43^,^44^,^46^. Briefly, an excess infrared spectrum is defined as the difference between the spectrum of a real solution and that of the respective ideal solution under identical conditions. The working equation in calculating the excess infrared spectrum of a binary system is as follows:


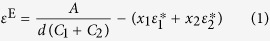


where *A* is the absorbance of the mixture, *d* is the light path length or the penetration depth in the case of ATR measurements, *C*_1_ and *C*_2_ are molarities of the two components, *x*_1_ and *x*_2_ are mole fractions of components 1 and 2, and 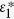 and 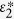 are molar absorption coefficients of the two components in their pure states, respectively.

### Quantum Chemical Calculations

All calculations were carried out using the Gaussian 09 package^53^. The geometries (isolated single molecules, their self-associates and complexes) were first optimized in gas phase using B3LYP method with 6–31++G** basis set. Then the energy, vibrational frequency, electrostatic surface potential and the natural population analysis (NPA) charge were calculated at the same level. The B3LYP functional has been successfully and extensively used to study the hydrogen bonding interactions in amide-containing systems^3^,^12^,^17^,^54^,^55^,^56^,^57^.

**Figure 4:**
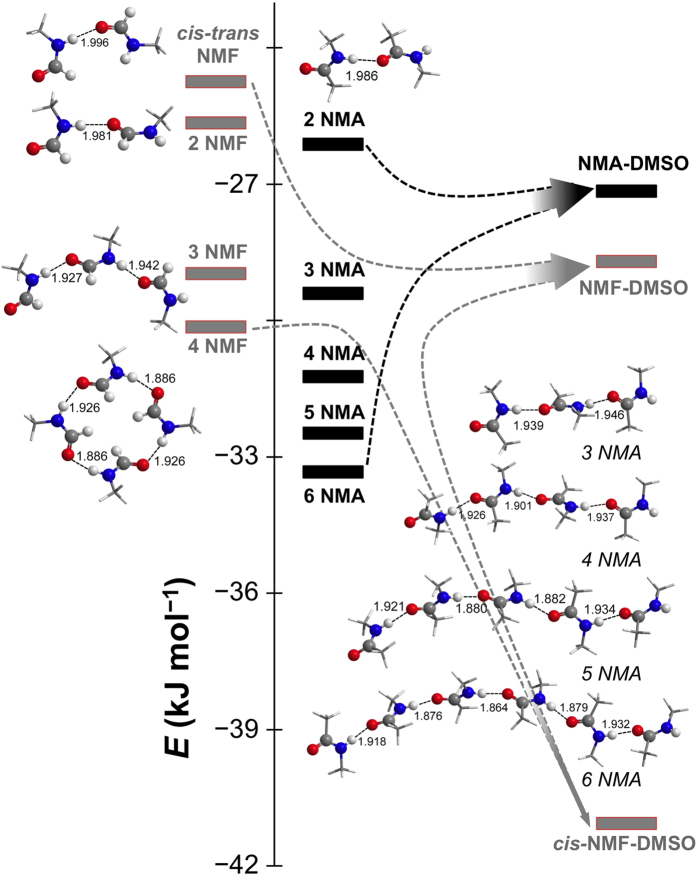
Calculated average relative hydrogen bond energies *E* and configurations of different complexes. Dashed arrows indicate the energy change of different complexes with adding DMSO to NMF (grey color) or NMA (black color). In the configurations, only −N−H···O = C− units are drawn in the ball-stick model, and N, H, O, C atoms are shown in blue, white, red, and grey balls, respectively. The numbers labeled in the models are the hydrogen bond lengths, and the unit is Å. All the NMF are *trans*-conformers without specific notification.

**Figure 5:**
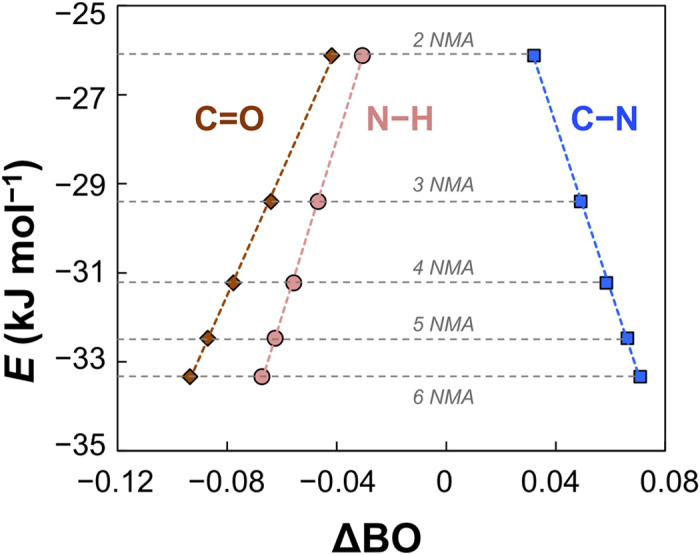
The relationship between average hydrogen bond energy *E* and average bond order change ΔBO of C = O, N−H and C−N in different NMA self-associates, taking the bond orders of the respective bonds in NMA monomer as references.

Electrostatic surface potentials of NMF, NMA, and DMF are calculated first to determine the possible hydrogen bonding sites between molecules. Then we searched possible structures for dimers of NMA, NMF and DMA. Totally 21 conformers were optimized and 9 stable conformers were obtained. Among the 9 stable conformers, we picked up the most stable conformers to represent the dimers of NMA, NMF and DMA. Their energies are shown in Fig. 4. For NMF, in agreement with the work of Cordeiro^41^, the most stable self-associating structures are dimer and trimer, and we repeated their calculation. For NMA, we added a single NMA to an optimized NMA dimer, and changed the position of the NMA. We have optimized 7 conformers, and 4 stable conformers were obtained. Similar method was applied to the calculation of tetramer and other self-associating structures. The primary structure of an oligomer was created by adding a monomer to the (*n* − 1) oligomer structure and then optimized by Gaussian.

Average hydrogen bond energy *E* was calculated by the interaction energy of each complexes divided by the number of hydrogen bonds. Energy zero points were set at the energies of individual molecules. All the optimized geometries were recognized as local energy minima with no imaginary frequency. The natural population analysis was done with the nature bond orbital (NBO) method^44^. Meanwhile, the basis set superposition error (BSSE) correction^58^ was performed for obtaining accurate interaction energies.

## Additional Information

**How to cite this article**: Zhou, Y. *et al*. Evidences for Cooperative Resonance-Assisted Hydrogen Bonds in Protein Secondary Structure Analogs. *Sci. Rep.* **6**, 36932; doi: 10.1038/srep36932 (2016).

**Publisher’s note:** Springer Nature remains neutral with regard to jurisdictional claims in published maps and institutional affiliations.

## Supplementary Material

Supplementary Information
