# Supplementary material for: Evidences for Cooperative Resonance-Assisted Hydrogen Bonds in Protein Secondary Structure Analogs
Source: Sci Rep. 2016 Nov 16;6:36932. doi: 10.1038/srep36932 (PMC5111121; doi:10.1038/srep36932)
Supplement: Supplementary Information [file srep36932-s1.doc]

**Supporting Information for**

**“Evidences for Cooperative Resonance-Assisted Hydrogen Bonds in Protein Secondary Structure Analogs”**

Yu Zhou‡, Geng Deng‡, Yanzhen Zheng, Jing Xu, Hamad Ashraf, Zhiwu Yu*

*Key Laboratory of Bioorganic Phosphorous Chemistry and Chemical Biology (Ministry of Education), Department of Chemistry, Tsinghua University, Beijing 100084, P. R. China*

*‡These authors contributed equally.*


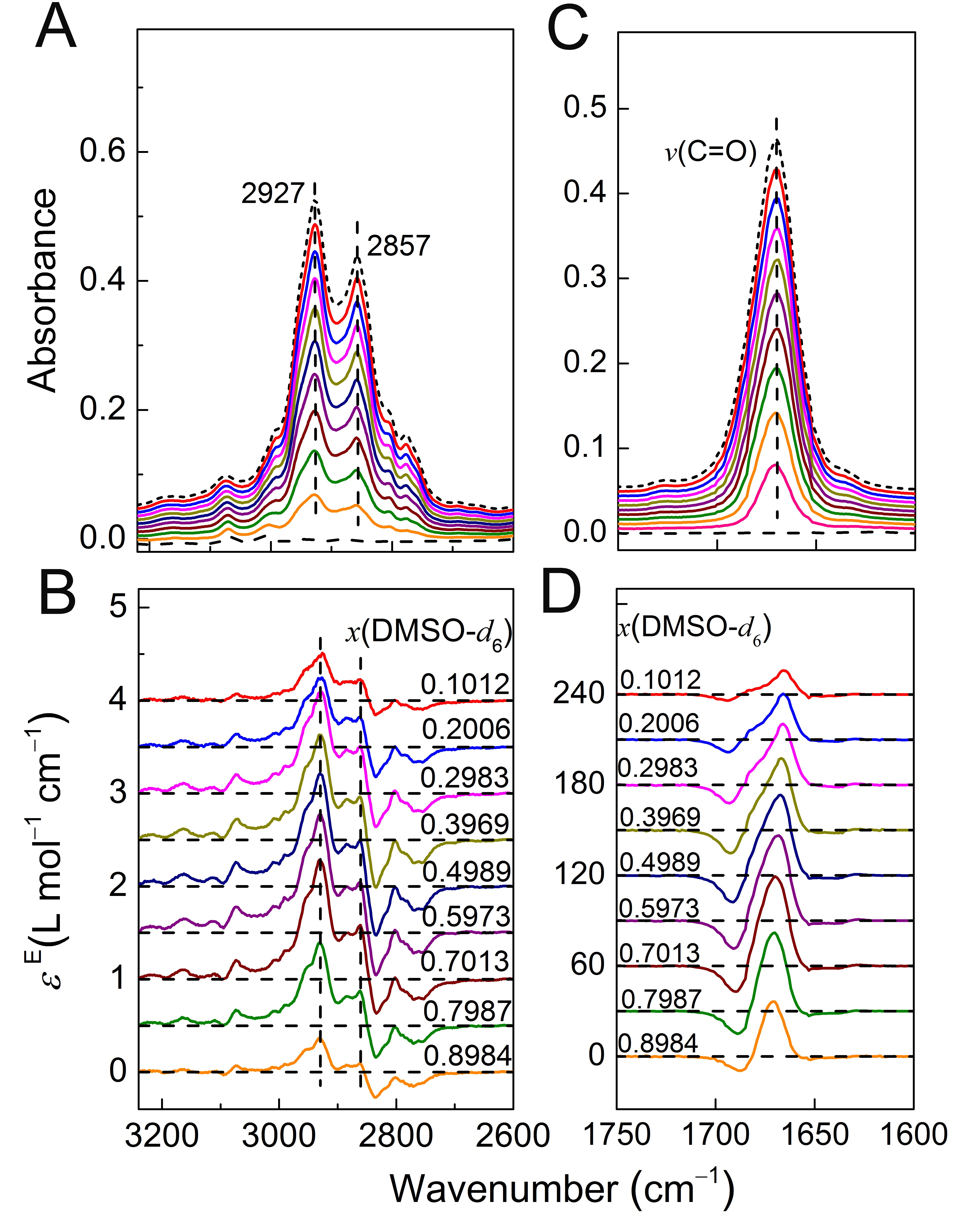


**Figure S1.** The IR (A and C) and excess IR (B and D) spectra of DMF-DMSO-*d*_6_ system in C−H (A and B) and C=O (C and D) stretching vibration regions. The short-dashed line and dashed line in (A, C) depict the spectra of pure DMF and DMSO-*d*_6_. The vertical dashed lines are used to guide eyes. The horizontal dashed lines in (B, D) are relative baselines for corresponding excess IR spectra. From top to bottom in (A, C), the mole fraction of DMSO-*d*_6_ increases from 0 to 1 with an increment of about 0.1. The precise mole fractions are labeled in (B) and (D). The nearly fixed C=O peak position in (C) shows very weak interaction between DMF and DMSO.


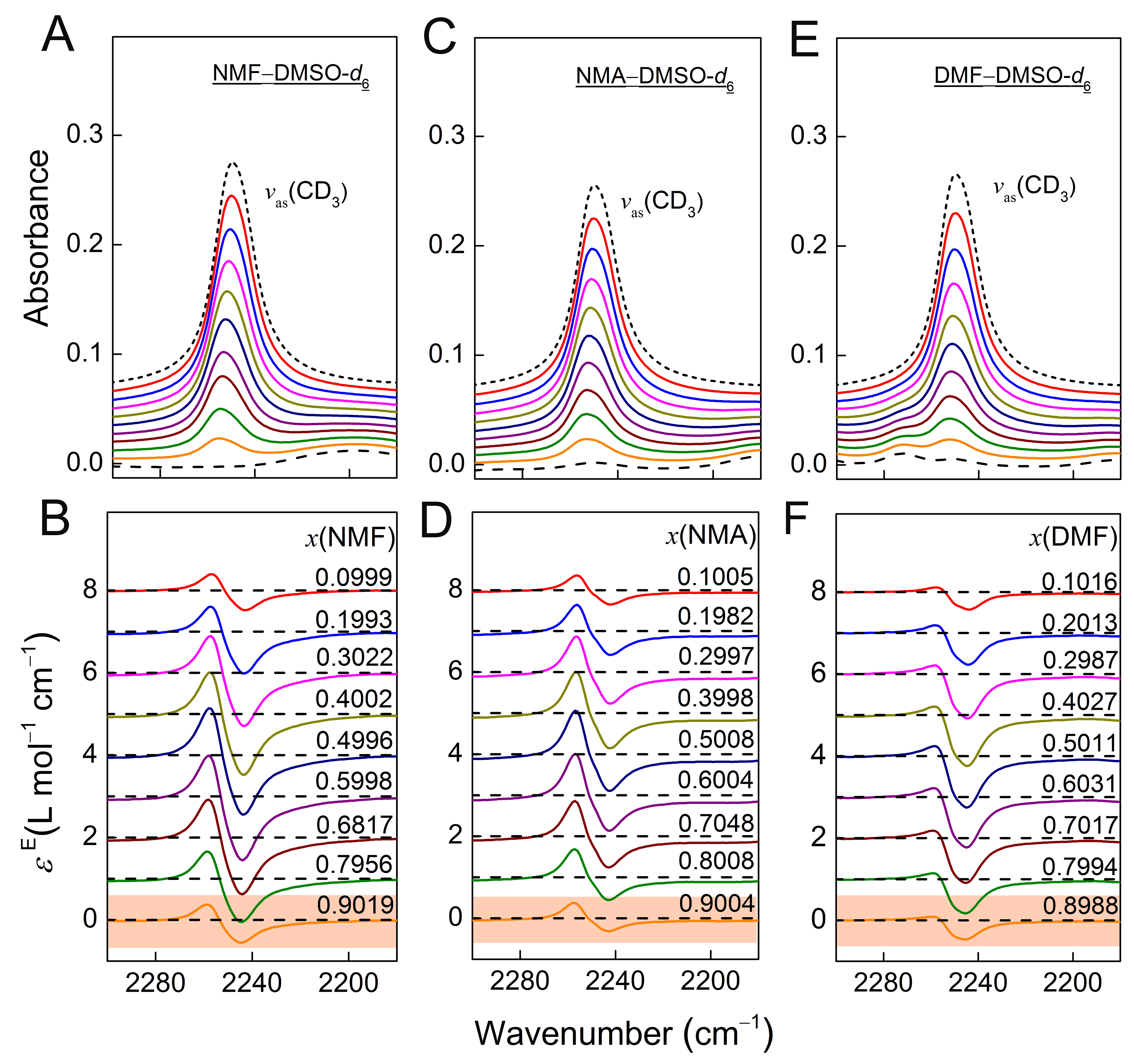


**Figure S2.** The IR (A, C and E) and excess IR (B, D and F) spectra of NMF (A and B)/NMA (C and D)/DMF (E and F)-DMSO-*d*_6_ system in C−D stretching vibration region. The short-dashed line and dashed line in (A, C, E) depict the spectra of pure NMF/NMA/DMF and DMSO-*d*_6_. The vertical dashed lines are used to guide eyes. The horizontal dashed lines in (B, D, F) are relative baselines for corresponding excess IR spectra. From top to bottom in (A, C, E), the mole fraction of DMSO-*d*_6_ increases from 0 to 1 with an increment of about 0.1. The precise mole fractions are labeled in (B), (D) and (F).


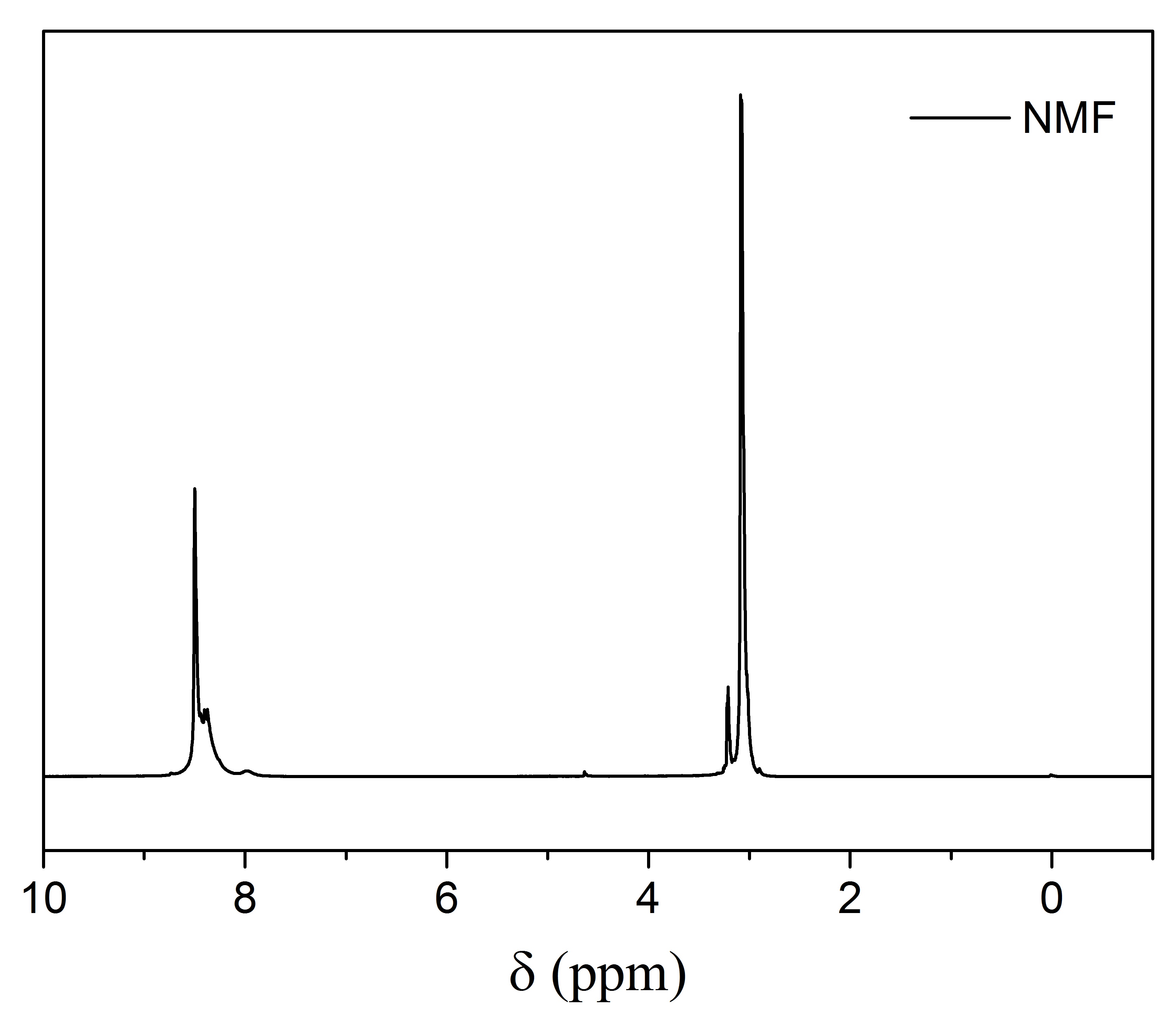


**Figure S3.** The ^1^H NMR spectrum of pure NMF. *δ* = 3.01 ppm is the peak of the methyl group in *trans*-NMF. *δ* = 3.20 ppm is the peak of the methyl group in *cis*-NMF. The ratio of the integral area is 9.43:1. Thus, *cis*-NMF is the minor component (~10%).


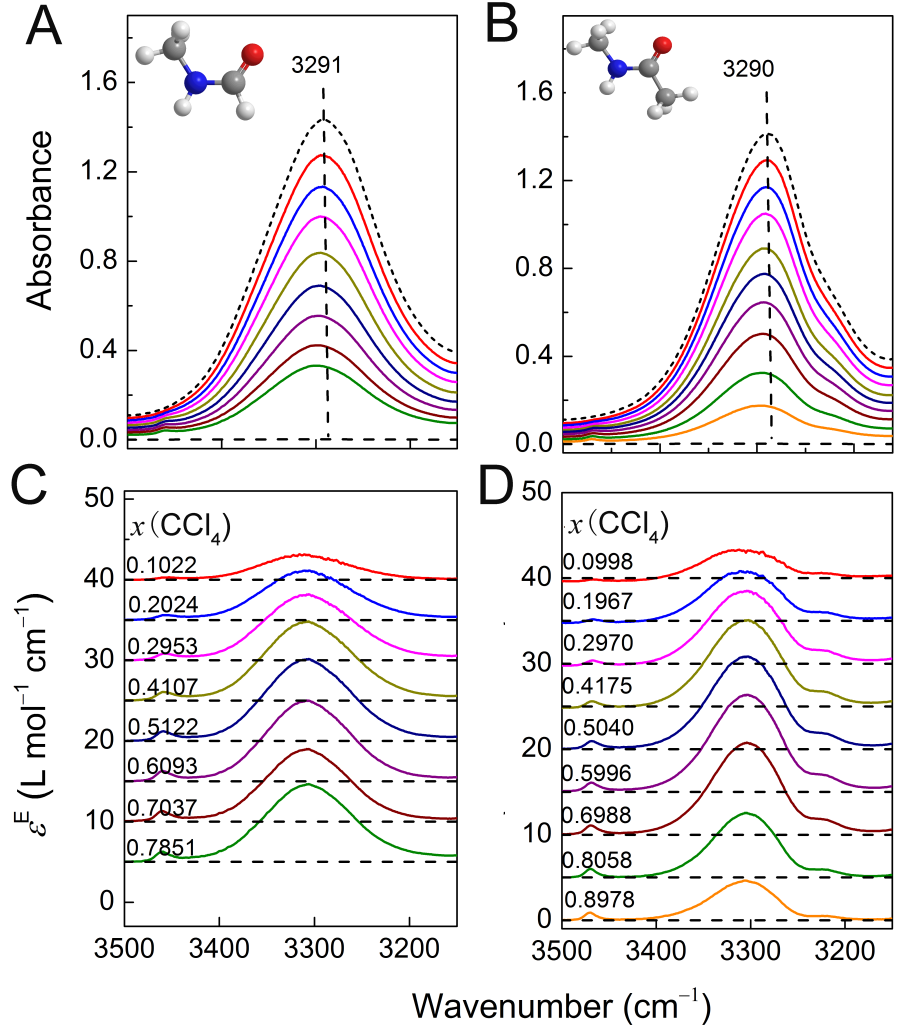


**Figure S4.** The IR (A, B) and excess IR (C, D) spectra of NMF-CCl_4_ (A, C) and NMA- CCl_4_ (B, D) systems in the N−H and C−H stretching vibration region. The short-dashed line and dashed line in (A, B) depict the spectra of pure NMF/NMA and CCl_4_. The vertical dashed lines are used to guide eyes. The horizontal dashed lines in (C, D) are relative baselines for corresponding excess IR spectra. From top to bottom in (A, B), the mole fraction of CCl_4_ increases from 0 to 1. The precise mole fractions are labeled in (C) and (D). The blue shifts of *ν*(N−H) shown in the figure indicate the weakening of the hydrogen bonding interactions among the amide molecules in both systems.


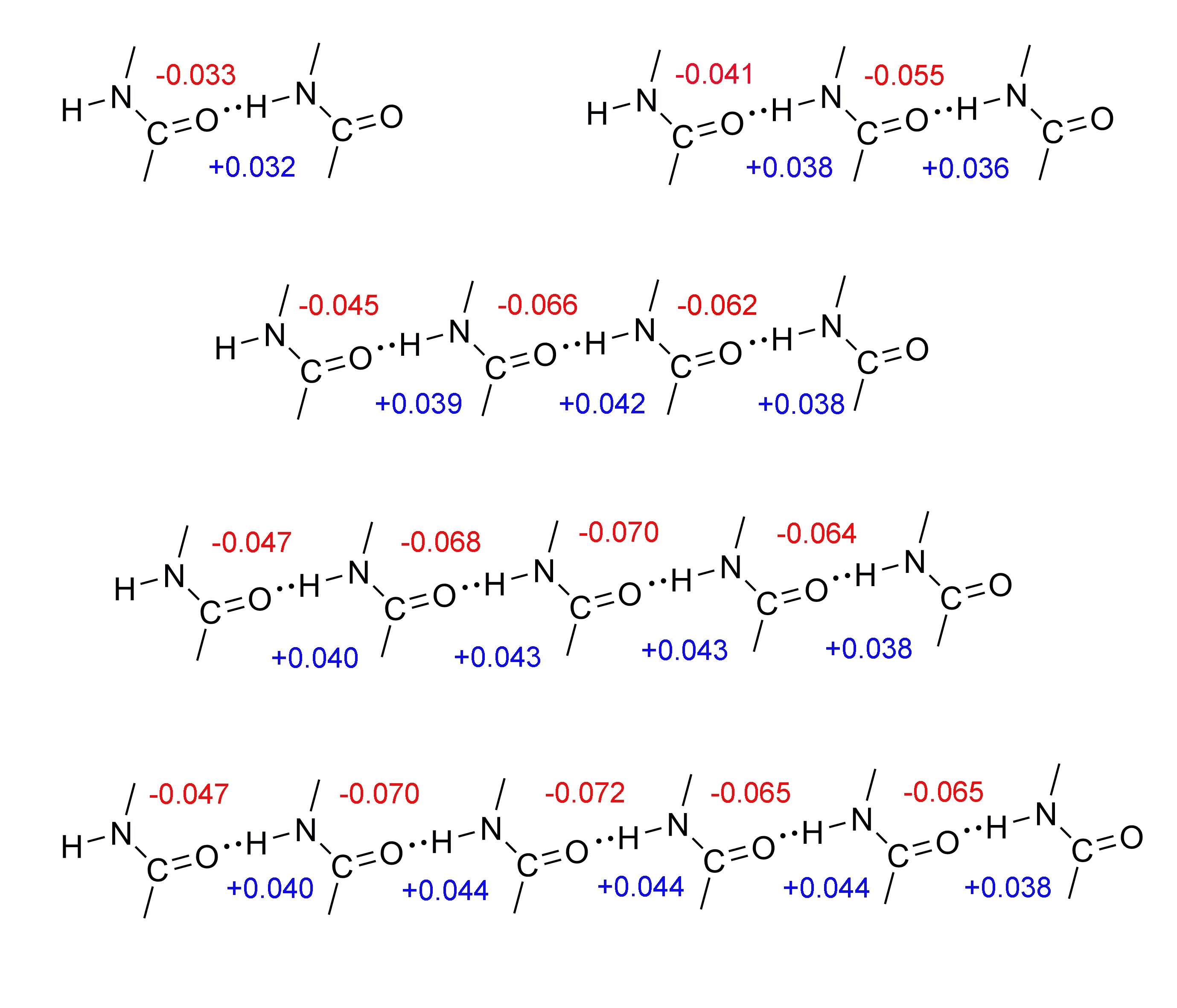


**Figure S5.** The NBO charge distributions in different self-associating structures of NMA. The results support our explanations on the charge change of resonance-assisted hydrogen bonds.

**
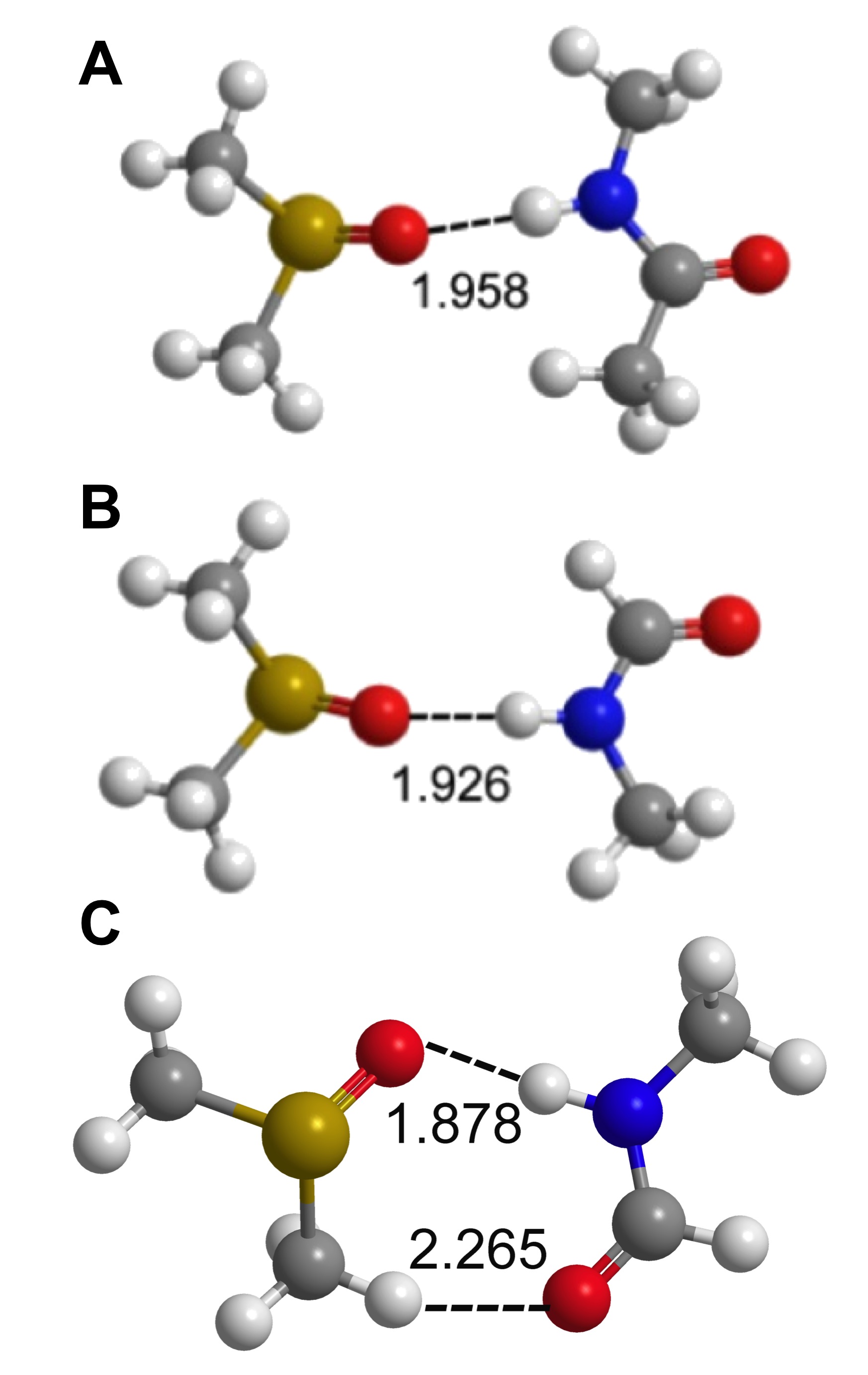
**

**Figure S6.** The optimized structures of NMA-DMSO (A), *trans*-NMF-DMSO (B) and *cis*-NMF-DMSO (C). Labeled in the figure are the hydrogen bond lengths.


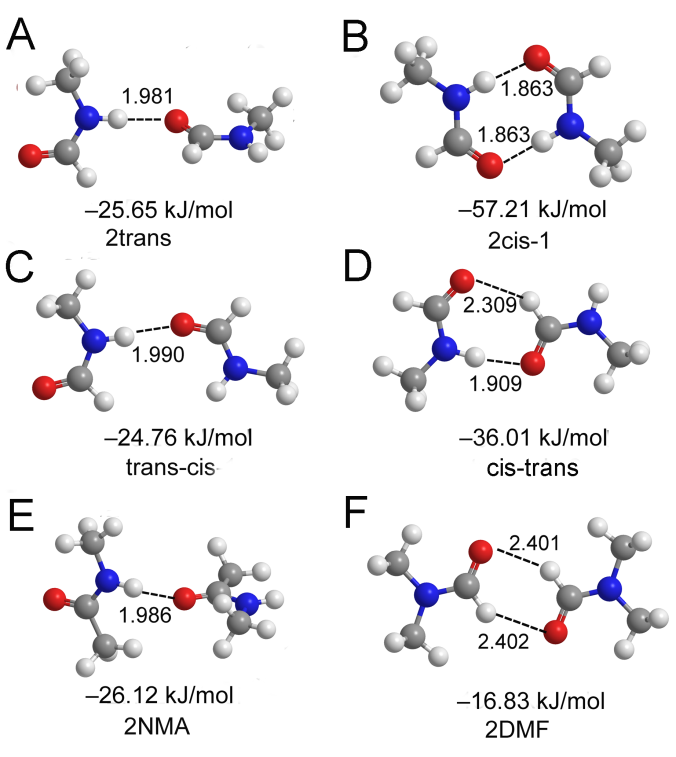


**Figure S7.** The optimized structures of NMF dimers. (A) 2trans-conformers, (B) 2cis-conformers, (C) trans-cis, and (D) cis-trans. Hydrogen bonds are denoted by dashed lines and the corresponding H···O distances are labeled. The corresponding interaction energies are shown below the complexes.

As is seen in the figure, two *trans*-NMF, as well as a *trans*-NMF and a *cis*-NMF, can form stable dimers with a single hydrogen bond (Figure S7A, S7C), the hydrogen bond distances and energies are quite similar. Two *cis*-NMF, as well as a *trans*-NMF and a *cis*-NMF, can form stable dimers with a ring structure (Figure S7B, S7D). Of which, the *cis*-NMF dimer is the most stable. The total interaction energy including the two hydrogen bonds (57.21 kJ/mol) is even more than twice those dimers when there is a single hydrogen bond ((25.65 and 24.76 kJ/mol)). It should be stressed that, in this case, both N−H and C=O in *cis*-NMF participate the formation of hydrogen bonds. Clearly, the formation of the ring structure hinders the extension of the chain-like structures. This conclusion is in agreement with that of the literature^[1]^.

**Reference**

(1) Martínez, A. G.; Vilar, E. T.; Fraile A. G. & Martıínez-Ruiz, P. Density functional theory study of self-association of *N*-methylformamide and its effect on intramolecular and intermolecular geometrical parameters and the *cis*/*trans* population. *J. Chem. Phys.* **124**, 234305 (2006).

**Table S1**. Single point energies of amides and DMSO

|  | *E* (a. u.) |
| --- | --- |
| *cis*-NMF | -209.2206704 |
| *trans*-NMF | -209.2221454 |
| NMA | -248.5488412 |
| DMF | -248.5335306 |
| DMSO | -553.2085086 |

**Table S2**. Single point energies of amide-amide and amide-DMSO complexes

|  | *E* (a. u. ) | *E*_BSSE corrected_ (a. u. ) | Δ*E* (a. u. ) | Δ*E* (kJ mol^-1^) | Δ*E*_average_ (kJ mol^-1^) |
| --- | --- | --- | --- | --- | --- |
| 2 NMA | -497.0976824 | -497.1076322 | -0.00994985 | -26.12 | -26.12 |
| 3 NMA | -745.6465235 | -745.6689151 | -0.022391527 | -58.79 | -29.39 |
| 4 NMA | -994.1953647 | -994.2310495 | -0.035684739 | -93.69 | -31.23 |
| 5 NMA | -1242.800368 | -1242.797762 | -0.053555968 | -129.90 | -32.48 |
| 6 NMA | -1491.293047 | -1491.356513 | -0.063466266 | -166.63 | -33.33 |
| *cis*-*trans* NMF | -418.4428158 | -418.4522392 | -0.009423412 | -24.74 | -24.74 |
| 2 NMF | -418.4442908 | -418.4540614 | -0.009770561 | -25.65 | -25.65 |
| 3 NMF | -627.6664362 | -627.6885062 | -0.022069928 | -57.94 | -28.97 |
| 4 NMF | -836.8885816 | -836.9344508 | -0.045869135 | -120.43 | -30.1 |
| *cis*-NMF-DMSO | -762.429179 | -762.4448236 | -0.015644585 | -41.07 |  |
| *trans*-NMF-DMSO | -762.430654 | -762.4415858 | -0.010931781 | -28.70 |  |
| NMA-DMSO | -801.7573498 | -801.7676911 | -0.010341336 | -27.15 |  |

**Table S3**. Bond lengths of C=O, N−H and C−N in amides and the concerned complexes

|  | *d*(C=O)/Å | *d*(N−H)/Å | *d*(C−N)/Å |
| --- | --- | --- | --- |
| *trans*-NMF | 1.223 | 1.01 | 1.361 |
| *cis*-NMF | 1.222 | 1.013 | 1.36 |
| 2 NMF | 1.228 | 1.018 | 1.354 |
|  | 1.229 | 1.01 | 1.353 |
| 3 NMF | 1.23 | 1.022 | 1.35 |
|  | 1.236 | 1.019 | 1.345 |
|  | 1.23 | 1.011 | 1.351 |
| 4 NMF | 1.24 | 1.024 | 1.338 |
|  | 1.24 | 1.027 | 1.339 |
|  | 1.24 | 1.024 | 1.338 |
|  | 1.24 | 1.027 | 1.339 |
| *cis-trans*-NMF | 1.228 | 1.015 | 1.351 |
|  | 1.228 | 1.013 | 1.352 |
| NMA | 1.229 | 1.008 | 1.366 |
| 2 NMA | 1.234 | 1.016 | 1.358 |
|  | 1.236 | 1.008 | 1.357 |
| 3 NMA | 1.235 | 1.019 | 1.357 |
|  | 1.242 | 1.018 | 1.35 |
|  | 1.237 | 1.008 | 1.356 |
| 4 NMA | 1.235 | 1.02 | 1.356 |
|  | 1.243 | 1.021 | 1.348 |
|  | 1.243 | 1.018 | 1.348 |
|  | 1.238 | 1.008 | 1.355 |
| 5 NMA | 1.236 | 1.02 | 1.356 |
|  | 1.234 | 1.022 | 1.347 |
|  | 1.245 | 1.022 | 1.346 |
|  | 1.244 | 1.018 | 1.347 |
|  | 1.238 | 1.008 | 1.355 |
| 6 NMA | 1.236 | 1.02 | 1.356 |
|  | 1.244 | 1.022 | 1.347 |
|  | 1.025 | 1.023 | 1.345 |
|  | 1.246 | 1.022 | 1.345 |
|  | 1.244 | 1.019 | 1.347 |
|  | 1.238 | 1.008 | 1.355 |
| *trans*-NMF-DMSO | 1.229 | 1.02 | 1.35 |
| *cis*-NMF-DMSO | 1.233 | 1.028 | 1.347 |
| NMA-DMSO | 1.234 | 1.017 | 1.358 |

**Table S4**. Bond orders of C=O, N−H, C−N and O···H in NMA complexes

|  | C=O | C−N | N−H | O···H |
| --- | --- | --- | --- | --- |
| **NMA** | | | | |
|  | 1.6575 | 1.1968 | 0.7942 |  |
| **2 NMA** | | | | |
| 1 | 1.6323 | 1.2256 | 0.7367 | 0.0326 |
| 2 | 1.5993 | 1.2322 | 0.7904 |  |
| Average | 1.6158 | 1.2289 | 0.7636 |  |
| **3 NMA** | | | | |
| 1 | 1.6256 | 1.2325 | 0.7254 | 0.0405 |
| 2 | 1.5673 | 1.2652 | 0.7276 | 0.0371 |
| 3 | 1.5876 | 1.2400 | 0.7893 |  |
| Average | 1.5935 | 1.2459 | 0.7474 | 0.0388 |
| **4 NMA** | | | | |
| 1 | 1.6232 | 1.2341 | 0.7224 | 0.0422 |
| 2 | 1.5588 | 1.2717 | 0.7168 | 0.0442 |
| 3 | 1.5541 | 1.2730 | 0.7260 | 0.0373 |
| 4 | 1.5833 | 1.2423 | 0.7888 |  |
| Average | 1.5799 | 1.2553 | 0.7385 | 0.0412 |
| **5 NMA** | | | | |
| 1 | 1.6222 | 1.2355 | 0.7213 | 0.0428 |
| 2 | 1.5550 | 1.2765 | 0.7111 | 0.0489 |
| 3 | 1.5444 | 1.2830 | 0.7121 | 0.0475 |
| 4 | 1.5492 | 1.2768 | 0.7253 | 0.0374 |
| 5 | 1.5816 | 1.2432 | 0.7887 |  |
| Average | 1.5705 | 1.2630 | 0.7317 | 0.0442 |
| **6 NMA** | | | | |
| 1 | 1.6216 | 1.2357 | 0.7202 | 0.0437 |
| 2 | 1.5536 | 1.2772 | 0.7106 | 0.0487 |
| 3 | 1.5403 | 1.2863 | 0.7067 | 0.0520 |
| 4 | 1.5399 | 1.2856 | 0.7112 | 0.0479 |
| 5 | 1.5470 | 1.2771 | 0.7244 | 0.0382 |
| 6 | 1.5808 | 1.2438 | 0.7886 |  |
| Average | 1.5639 | 1.2676 | 0.7270 | 0.0461 |

**Table S5**. Atom coordinates of amide-amide and amide-DMSO complexes

| **2 NMA** |
| --- |
| C -2.12167000 0.25518600 0.36866300  O -1.15024200 -0.04211200 -0.33509000  N -3.38322700 -0.09352400 0.00976700  H -4.15283900 0.16974900 0.60554900  C -3.65741400 -0.83143700 -1.21780900  H -3.12072200 -1.78429500 -1.22174200  H -4.73028000 -1.02116600 -1.27930700  H -3.34064200 -0.25845800 -2.09430200  C -1.96824800 1.01957400 1.66967400  H -2.91801300 1.22155500 2.17257900  H -1.32498900 0.44604700 2.34308800  H -1.46506500 1.96867000 1.46532400  C 2.70404200 -0.47906800 0.09111800  O 3.92174500 -0.28176200 0.08121800  N 1.81220400 0.44073000 -0.36033000  H 0.81605400 0.23998700 -0.33717300  C 2.25989000 1.71162100 -0.90513600  H 2.93943600 1.55845700 -1.75013400  H 1.38593800 2.27176200 -1.24480400  H 2.79594400 2.29958600 -0.15191900  C 2.11138400 -1.77872100 0.61494300  H 2.41679200 -1.90974300 1.65747800  H 1.02070900 -1.81408300 0.54720700  H 2.53491100 -2.61045700 0.04435100 |
| **3 NMA** |
| C 4.59331100 0.03671300 -0.30780400  O 3.45405800 -0.12720700 0.14504700  N 5.52034500 -0.95134600 -0.25568400  H 6.43039900 -0.78865800 -0.65861100  C 5.22611600 -2.25912300 0.31911000  H 4.52916700 -2.82115300 -0.31104000  H 6.15812600 -2.81906500 0.41204100  H 4.77448200 -2.14054500 1.30682100  C 5.02455800 1.34652500 -0.93817900  H 6.05283700 1.33514400 -1.30963200  H 4.34936400 1.57965300 -1.76617700  H 4.92499200 2.14497300 -0.19728400  C -0.30424600 0.50126100 0.09006700  O -1.45303000 0.90660000 0.33054200  N 0.78042900 1.04077300 0.68529300  H 1.70498900 0.66967600 0.47920400  C 0.65359700 2.11645900 1.65692100  H 0.06506400 1.79759400 2.52354700  H 1.65336500 2.40311500 1.98876100  H 0.15499100 2.98516900 1.21568200  C -0.04503300 -0.62378800 -0.89493900  H -0.39313300 -0.31483800 -1.88550000  H 1.00960200 -0.90283600 -0.95314500  H -0.63574500 -1.49559900 -0.59962100  C -4.95161500 -0.68081000 0.20382400  O -6.08837900 -1.01696300 -0.14221100  N -4.17133000 0.13788800 -0.54599400  H -3.23704700 0.39034700 -0.22632400  C -4.65261500 0.68202700 -1.80400200  H -5.57098800 1.26064100 -1.65672000  H -3.88040800 1.33417700 -2.21800700  H -4.87631900 -0.11483400 -2.52221600  C -4.34070000 -1.16727600 1.50962000  H -4.30524700 -2.26098500 1.49554600  H -3.33779100 -0.77127200 1.68952900  H -4.99824600 -0.87329100 2.33310300 |
| **4 NMA** |
| C 2.16572800 0.33831700 0.34884400  O 0.96442000 0.26345100 0.65889100  N 3.10123000 -0.43702200 0.93262100  H 4.07482200 -0.35304400 0.64629100  C 2.75350700 -1.39586300 1.97084500  H 2.10968100 -2.19050000 1.57872600  H 3.67428900 -1.83733700 2.35663800  H 2.21895800 -0.90408100 2.78898400  C 2.65581700 1.31266800 -0.70563900  H 3.73487100 1.25748100 -0.86625300  H 2.13591600 1.10888700 -1.64659300  H 2.39046100 2.32838200 -0.39700900  C -2.69239000 0.43537900 -0.11839900  O -3.85383000 0.80152100 -0.36860600  N -1.67580300 1.31479500 -0.01282000  H -0.73572100 0.97918300 0.20001300  C -1.89507800 2.74338500 -0.17427500  H -2.62057800 3.11483500 0.55680000  H -0.94340900 3.25830000 -0.02851000  H -2.28131200 2.97271600 -1.17299900  C -2.34332100 -1.02756400 0.08300000  H -2.59791300 -1.58079400 -0.82633600  H -1.28825400 -1.18217300 0.31967100  H -2.96041300 -1.42943600 0.89213800  C -7.41601600 -0.43490000 0.28213200  O -8.53434000 -0.94664900 0.16551300  N -6.48918500 -0.46905900 -0.70782600  H -5.57494400 -0.03886400 -0.56939300  C -6.77829500 -1.10745900 -1.97990800  H -7.67196000 -0.67410100 -2.44153400  H -5.92366900 -0.96057100 -2.64410500  H -6.95882600 -2.18159400 -1.85620200  C -6.99579500 0.27819900 1.55876400  H -7.05417700 -0.42875300 2.39214900  H -5.98758300 0.69694100 1.50470500  H -7.71113400 1.08082900 1.76145300  C 6.89220200 -0.69942400 -0.39481900  O 5.92575100 -0.12433600 0.12189100  N 8.08223400 -0.07035800 -0.55027600  H 8.84368200 -0.56616100 -0.98770100  C 8.28913300 1.30968100 -0.12518000  H 8.03650700 1.42458700 0.93214300  H 9.33836200 1.56809200 -0.27615000  H 7.66221000 1.99529000 -0.70336800  C 6.80775600 -2.13389900 -0.87794000  H 5.99387800 -2.21573900 -1.60364800  H 7.73166500 -2.49482800 -1.33772000  H 6.55975100 -2.77904400 -0.03014500 |
| **5 NMA** |
| C 5.07157800 -0.67762400 0.36434300  O 6.21795400 -0.46222900 0.79595400  N 4.05131900 -1.00723700 1.18017200  C 4.24479400 -1.14353100 2.61501300  C 4.74757200 -0.58349600 -1.11548400  H 3.11964300 -1.16686500 0.79085900  H 4.54617400 -0.19263900 3.06796800  H 3.30373200 -1.47264800 3.06001000  H 5.02502500 -1.87904000 2.83528900  H 3.70477900 -0.82275800 -1.33589400  H 4.97267200 0.42866700 -1.46552100  H 5.39930400 -1.27049400 -1.66352500  C 9.25917800 1.65707800 -0.10968600  O 10.34954500 2.04957000 -0.53816500  N 8.81269100 0.39009300 -0.29570100  C 9.61767300 -0.58911000 -1.00479300  C 8.33036600 2.57881600 0.66693200  H 7.90486400 0.10708900 0.07345600  H 10.58930500 -0.72722600 -0.51792200  H 9.08119700 -1.54051200 -1.01048000  H 9.80797500 -0.27514800 -2.03737200  H 8.08155100 3.43898900 0.03770500  H 7.41103300 2.08557800 0.99303000  H 8.86742800 2.95872000 1.54122500  C 0.30824600 -0.97199100 -0.01147400  O 1.41625500 -1.53166900 0.08396800  N -0.74433900 -1.57328500 -0.59615400  C -0.63462400 -2.90753800 -1.16553700  C 0.07466500 0.42622700 0.52890400  H -1.63669100 -1.08001900 -0.66313900  H -0.35992000 -3.64129300 -0.40065400  H -1.60000500 -3.17893000 -1.59679100  H 0.13023600 -2.93863600 -1.94825500  H 0.80035000 1.10659000 0.07348900  H -0.93599500 0.79145500 0.33337200  H 0.25935500 0.42587000 1.60774100  C -9.09103900 1.05927400 0.47646100  O -8.13287700 0.47910000 -0.05023400  N -10.27067400 0.42453500 0.67948500  C -10.47637200 -0.96810500 0.29705700  C -9.00743800 2.50687200 0.91849500  H -11.02392900 0.92457300 1.12624700  H -9.84362300 -1.63543400 0.89033600  H -11.52376900 -1.22496500 0.46265900  H -10.23077900 -1.11206400 -0.75820900  H -8.78830600 3.13091300 0.04724700  H -9.92229200 2.87200000 1.39277700  H -8.17550100 2.61548400 1.61980200  C -4.42629300 -0.14113500 -0.49185400  O -3.24127200 -0.11932100 -0.86921800  N -5.34196100 0.71850200 -0.97935500  C -4.99288100 1.70857400 -1.98765500  C -4.91402800 -1.13736400 0.54269900  H -6.30402000 0.67001200 -0.64888200  H -4.56108800 1.22865300 -2.87116200  H -5.89974700 2.24327600 -2.27598000  H -4.25909900 2.42414200 -1.60219200  H -4.70779200 -2.14998400 0.18338200  H -4.34536200 -0.99614700 1.46691400  H -5.98118000 -1.04075200 0.75438700 |
| **6 NMA** |
| C 2.67165700 -1.59290500 0.20871000  O 3.85176500 -1.42179500 0.56828000  N 1.65047300 -1.57509200 1.08416600  C 1.87452100 -1.37003100 2.50667100  C 2.31167700 -1.83681300 -1.24480000  H 0.69223300 -1.70888600 0.75073200  H 2.26931000 -0.36801200 2.70816100  H 0.92235800 -1.49221500 3.02643400  H 2.59442300 -2.09686800 2.89518300  H 1.23813600 -1.96767100 -1.39806400  H 2.66731900 -0.99421900 -1.84570900  H 2.83603300 -2.73201300 -1.59296100  C 7.27116300 -0.17519500 -0.24135300  O 8.45949000 -0.07131400 -0.59477700  N 6.55997200 -1.29991700 -0.45135500  C 7.15729800 -2.46284000 -1.08800700  C 6.54360600 0.96364300 0.44915600  H 5.58933100 -1.35297400 -0.13506800  H 8.01938400 -2.82433400 -0.51764300  H 6.40512500 -3.25244700 -1.13997400  H 7.50316800 -2.22465600 -2.09933700  H 6.53773900 1.83433200 -0.21401400  H 5.51776500 0.70583800 0.72154700  H 7.09924500 1.24334500 1.34920100  C 11.53380900 1.90657700 0.52099100  O 12.43680000 2.74542900 0.60929700  N 10.56067900 1.97506100 -0.42117000  C 10.54169500 3.04891700 -1.39859200  C 11.44911800 0.72383400 1.47440100  H 9.83064000 1.26298600 -0.45748900  H 11.47209100 3.07144600 -1.97646600  H 9.70167800 2.88530900 -2.07730700  H 10.43039700 4.02607800 -0.91475000  H 11.36268400 1.10212300 2.49771400  H 10.60903600 0.05883600 1.25831800  H 12.38391200 0.15859400 1.41346500  C -2.09849900 -1.37504100 0.00609000  O -1.04717900 -2.02551100 0.15970400  N -3.23570000 -1.95344300 -0.42098900  C -3.29272000 -3.37240700 -0.73686200  C -2.16286200 0.11299100 0.29346700  H -4.07702800 -1.38538500 -0.53960700  H -3.06658400 -3.98246100 0.14394800  H -4.29865600 -3.60740500 -1.08963600  H -2.56902700 -3.62998500 -1.51674700  H -1.40379400 0.62263700 -0.30750200  H -3.14383700 0.54155600 0.07775200  H -1.91458700 0.28244600 1.34593600  C -11.15813900 1.85194400 0.40687200  O -10.26923800 1.09596200 -0.00637200  N -12.38659400 1.38279300 0.73276300  C -12.73309300 -0.03006300 0.62607400  C -10.93367500 3.34330800 0.56177700  H -13.07541100 2.02768800 1.08881000  H -12.20591800 -0.62477900 1.37905200  H -13.80890700 -0.13866200 0.77234400  H -12.46220700 -0.40883700 -0.36228600  H -10.71837000 3.77282600 -0.42116300  H -11.78760400 3.87047600 0.99549300  H -10.05641100 3.50581300 1.19369700  C -6.72921900 -0.09207400 -0.47720200  O -5.53807100 -0.23459700 -0.80694400  N -7.46512400 0.95062500 -0.90882200  C -6.90649000 1.96090900 -1.79509900  C -7.43043500 -1.08834200 0.42609200  H -8.43386900 1.04063200 -0.60690300  H -6.45922600 1.49478200 -2.67772400  H -7.71027500 2.62941300 -2.10914500  H -6.12841800 2.54597900 -1.29272000  H -7.42696300 -2.06876200 -0.06039000  H -6.86503600 -1.18215800 1.35796800  H -8.46082200 -0.80445100 0.65087500 |
| ***cis*-*trans* NMF** |
| C 2.36572900 -1.02890000 -0.00961000  O 3.53872400 -1.39210900 0.00081800  N 1.94420500 0.25422300 -0.00206200  H 1.52661500 -1.75179700 -0.02702000  H 0.94599700 0.44101000 -0.01084600  C 2.88103700 1.36765100 0.02024300  H 3.54198300 1.33961100 -0.85224200  H 2.31320900 2.29955900 0.01214300  H 3.50793900 1.33449400 0.91750900  C -2.19663400 0.77126000 -0.01145200  O -0.98070700 0.94016300 -0.02462300  N -2.81215200 -0.43285100 0.00145500  C -4.25337000 -0.63474700 0.01743500  H -2.90261400 1.62106000 -0.00932400  H -2.20378300 -1.24312300 0.00010200  H -4.74817600 0.33955600 0.01648300  H -4.58421400 -1.18847900 -0.86711000  H -4.56603400 -1.17751100 0.91529300 |
| **2 NMF** |
| C -2.55998800 -0.87232800 -0.21437900  O -3.78099700 -1.00151500 -0.19612100  N -1.87571700 0.28059000 -0.02233800  H -1.88489700 -1.73046900 -0.39473900  H -0.85931600 0.24800900 -0.07655400  C -2.53193500 1.55614500 0.21404900  H -2.16666500 2.01420300 1.13962800  H -2.35449400 2.25126200 -0.61480500  H -3.60449000 1.37737800 0.30206200  C 1.93130100 -0.34934500 0.62974600  O 1.11994700 0.17544400 -0.13043800  N 3.27125300 -0.36129800 0.44604100  H 1.62050200 -0.86761000 1.55350700  H 3.83127400 -0.83582100 1.14050900  C 3.93602100 0.24813400 -0.69973800  H 4.67793900 0.98086000 -0.36826200  H 4.42982600 -0.51180500 -1.31379800  H 3.17758600 0.75188300 -1.29907400 |
| **3 NMF** |
| C -0.31715300 1.00943200 -0.04388400  O -1.47725800 1.43242500 -0.10677400  N 0.79519600 1.74543000 -0.21418800  H -0.09705700 -0.05114600 0.16572800  H 1.69110400 1.26615300 -0.13916700  C 0.76980200 3.16890800 -0.51501900  H 1.24391000 3.74552000 0.28687100  H 1.29535100 3.37237800 -1.45382000  H -0.27006200 3.48287500 -0.61117100  C 4.30482700 -0.11280200 0.55221700  O 3.24022300 0.10095700 -0.02609600  N 5.04169200 -1.23740800 0.42374000  H 4.75848500 0.62351700 1.23768700  H 5.88755000 -1.30431400 0.97262300  C 4.65378700 -2.36765500 -0.41325400  H 5.50589700 -2.69949000 -1.01218000  H 4.29233700 -3.20390400 0.19448900  H 3.85232400 -2.04110700 -1.07599300  C -4.13141500 -1.19960300 -0.77694600  O -5.04566500 -2.02041000 -0.72557000  N -3.79750200 -0.34000100 0.20917200  H -3.48398500 -1.09602100 -1.66862200  H -3.01462700 0.30058400 0.06477200  C -4.53179300 -0.30047900 1.46390600  H -4.47374400 -1.26201300 1.98573600  H -4.09957400 0.47826100 2.09523800  H -5.59033300 -0.07802100 1.29214500 |
| **4 NMF** |
| C -2.60257900 -0.78537200 0.07002600  O -2.81630100 -1.95492200 -0.28274300  N -3.50108800 0.20425000 0.13975500  H -1.59426200 -0.45728300 0.37668100  H -3.13562100 1.12716100 0.38923600  C -4.90097000 0.04397800 -0.22450800  H -5.53566900 0.52953900 0.52172600  H -5.10698000 0.48734800 -1.20541100  H -5.13408900 -1.02079200 -0.26349000  C -0.69573000 2.49734300 0.76005600  O -1.91998200 2.60641300 0.59854100  N 0.23783200 3.23455600 0.14537300  H -0.26703900 1.75334200 1.45460800  H 1.21959700 2.95874600 0.26951900  C -0.10650700 4.26485800 -0.82544000  H -0.82659200 4.96571400 -0.39485000  H 0.80286300 4.80254200 -1.09777100  H -0.55243100 3.83154800 -1.72782400  C 0.69601500 -2.49716100 -0.75978400  O 1.92025000 -2.60680900 -0.59847800  N -0.23773900 -3.23432200 -0.14535500  H 0.26752400 -1.75259500 -1.45385700  H -1.21945700 -2.95835800 -0.26952400  C 0.10637500 -4.26538100 0.82473400  H 0.55516400 -3.83297400 1.72611300  H -0.80362600 -4.80100400 1.09899500  H 0.82399600 -4.96793100 0.39274400  C 2.60249700 0.78570300 -0.06979500  O 2.81646500 1.95523300 0.28287700  N 3.50079800 -0.20410700 -0.13959100  H 1.59407000 0.45778600 -0.37629100  H 3.13512000 -1.12696200 -0.38899600  C 4.90076500 -0.04413400 0.22447400  H 5.10695900 -0.48825200 1.20499800  H 5.53531700 -0.52914200 -0.52225000  H 5.13388900 1.02060600 0.26419800 |
| ***cis*-NMF-DMSO** |
| C 2.41750600 -0.92194100 0.12092500  O 1.56380200 -1.80579700 0.21909700  N 2.16633100 0.39138900 -0.04508300  C 3.19584200 1.40857300 -0.15614100  H 3.49860700 -1.15968600 0.16552200  H 1.18054900 0.67783400 -0.09517700  H 4.18181700 0.94070100 -0.07941100  H 3.13613200 1.92808300 -1.11904400  H 3.10503100 2.15107300 0.64452300  S -1.64314900 0.12433300 0.44918800  O -0.64681400 1.09969700 -0.19321700  C -1.60751300 -1.39736500 -0.55941200  C -3.29492500 0.72389700 -0.05936800  H -0.60903200 -1.82105900 -0.42193200  H -2.37411300 -2.08648800 -0.19297300  H -1.77779600 -1.13624900 -1.60742300  H -4.05718800 0.01124500 0.26784600  H -3.45008500 1.68773400 0.42876700  H -3.30922500 0.84757700 -1.14518800 |
| ***trans*-NMF-DMSO** |
| C 2.80334200 0.89301900 -0.11585900  O 4.01642800 0.97637600 -0.29393000  N 2.12636200 -0.25005400 0.12637800  H 2.14275300 1.78101800 -0.14192800  H 1.11746600 -0.20533500 0.26699900  C 2.80665800 -1.53245600 0.21789300  H 3.55479400 -1.52399700 1.01772900  H 2.06515500 -2.30526000 0.42913000  H 3.32162700 -1.77196000 -0.71862000  S -1.92793000 0.11696700 -0.40942800  O -0.78657100 -0.20112000 0.55705300  C -2.92534500 1.42077600 0.40008300  C -3.13620800 -1.24586400 -0.22788500  H -2.30858900 2.32090800 0.42600800  H -3.83022000 1.60575000 -0.18535500  H -3.16711500 1.10472900 1.41797700  H -4.03517900 -1.01782000 -0.80722000  H -2.65916000 -2.14556900 -0.62066000  H -3.36872500 -1.37845100 0.83175700 |
| **NMA-DMSO** |
| C 2.77157600 -0.42834400 0.04205400  O 3.97339500 -0.25800300 -0.17961400  N 1.87649500 0.59314100 0.03500600  H 0.89239000 0.40946200 0.21607900  C 2.30282600 1.95811600 -0.22331900  H 2.72800300 2.05860900 -1.22818900  H 1.43633000 2.61680300 -0.13200500  H 3.06952400 2.27115300 0.49316400  C 2.20284600 -1.80720600 0.34204800  H 1.12857200 -1.79390800 0.54408600  H 2.40791800 -2.46468200 -0.50846400  H 2.72882600 -2.22051300 1.20761200  S -2.20329200 0.15440500 -0.40496300  O -1.00758500 0.06943100 0.54356100  C -3.50656600 1.05867900 0.50856200  C -2.99772800 -1.49450500 -0.37036200  H -3.15156700 2.08320900 0.63356400  H -4.43260300 1.05350500 -0.07305400  H -3.64775600 0.59098900 1.48617400  H -3.93336500 -1.46173600 -0.93545600  H -2.30266200 -2.19067000 -0.84320500  H -3.17061100 -1.78655200 0.66858600 |
